# Factors associated with the time to the first wheezing episode in infants: a cross-sectional study from the International Study of Wheezing in Infants (EISL)

**DOI:** 10.1038/npjpcrm.2015.77

**Published:** 2016-01-21

**Authors:** Rosa M Pacheco-Gonzalez, Javier Mallol, Dirceu Solé, Paul L P Brand, Virginia Perez-Fernandez, Manuel Sanchez-Solis, Luis Garcia-Marcos, the EISL Study Group

**Affiliations:** 1 Pediatric Respiratory and Allergy Unit, ‘Virgen de la Arrixaca’ University Children’s Hospital, University of Murcia, Murcia, Spain; 2 Department of Pediatric Respiratory Medicine, Hospital El Pino, University of Santiago de Chile (USACH), Chile, USA; 3 Division of Allergy, Clinical Immunology and Rheumatology, Department of Pediatrics, Federal University of São Paulo (UNIFESP), São Paulo, Brazil; 4 Princess Amalia Children’s Clinic, Isala Klinieken, Zwolle, The Netherlands; 5 UMCG Postgraduate School of Medicine, University Medical Centre Groningen, Groningen, The Netherlands; 6 Department of Pediatrics, University of Murcia, Murcia, Spain; 7 IMIB Bioresearch Institute, Murcia, Spain

## Abstract

Male gender, asthmatic heredity, perinatal tobacco smoke exposure and respiratory infections have been associated with wheeze in the first years of life, among other risk factors. However, information about what factors modify the time to the first episode of wheeze in infants is lacking. The present study analyses which factors are associated with shorter time to the first episode of wheeze in infants. Parents of 11- to 24-month-old children were surveyed when attending their health-care centres for a control visit. They answered a questionnaire including the age in months when a first wheeze episode (if any) had occurred (outcome variable). The study was performed in 14 centres in Latin America (LA) and in 8 centres in Europe (EU) (at least 1,000 infants per centre). Factors known to be associated with wheezing in the cohort were included in a survival analysis (Cox proportional hazards model). Summary hazard ratios adjusted for all risk factors (aHR) were calculated using the meta-analysis approach with random effects. A total of 15,067 infants had experienced wheezing at least once, out of 35,049 surveyed. Male gender in LA (aHR 1.05, 95% confidence interval (CI) 1.00–1.10, *P*=0.047), parental asthma in LA and EU (aHR 1.05, 95% CI 1.00–1.11, *P*=0.037), infant eczema in EU (aHR 1.25, 95% CI 1.12–1.39, *P*<0.001) and having a cold during the first 3 months in LA and EU (aHR 1.97, 95% CI 1.90–2.04, *P*<0.001), in LA (aHR 1.98, 95% CI 1.90–2.06, *P*<0.001) and in EU (aHR 1.91, 95% CI 1.75–2.09, *P*<0.001) were associated with a shorter period of time to the first episode. Breast feeding for at least 3 months was associated with a longer period, only in LA (aHR 0.91, 95% CI 0.86–0.96, *P*<0.001). Cold symptoms during the first 3 months is the most consistent factor shortening the time to the first episode of wheezing; breast feeding for ⩾3 months delays it only in LA, whereas eczema shortens it only in EU. Avoiding a common cold in the first months of life could be a good strategy to delay the first wheeze episode; however, cohort studies will help to elucidate this association.

## Introduction

Wheezing during the first months of life is a very frequent condition worldwide: 45% of infants who participated in the International Study of Wheezing in Infants (EISL, ‘Estudio Internacional de Sibilancias en Lactantes’) had at least one episode of wheezing and 23% had recurrent wheeze; however, these prevalences vary between localities.^1,2^ A considerable number of surveys have studied the factors associated with wheeze episodes during the first years of life; however, studies focused on the first 12 months of life are less numerous. Although not every study that focused on this first year of life addressed the same risk/protective factors or had the same results, some conclusions can be drawn: factors such as asthmatic heredity, male gender, perinatal tobacco smoke exposure, respiratory infections by common viruses, preterm birth, housing conditions, indoor air quality, outdoor air pollution, mould stains on household walls, Afro-American ethnicity and excess infant adiposity^3–14^ are common risk factors. Regarding protective factors, breast feeding is by far the most consistently reported one.^9,15^

Wheezing during the first months of life might be associated with later asthma, especially when episodes are severe and/or frequent.^14,16,17^ Time to the first episode of wheeze has been associated with the frequency of recurrence: the earlier the first episode occurs, the more frequently wheezing recurs.^18^ It is possible that some environmental factors could influence the innate immune response during the first months of life, a critical period for immune system development, resulting in more frequent and severe episodes of wheezing.^19^ However, no study has been performed to analyse which factors might be associated with the time to the first episode of wheeze.

In the present paper we perform a survival analysis and present the results of those factors associated with an earlier or a later episode of wheezing in a large cohort of 1-year-old infants from Latin America (LA) and Europe (EU; EISL or International Study of Wheezing in Infants).

## Results

**Figure 1:**
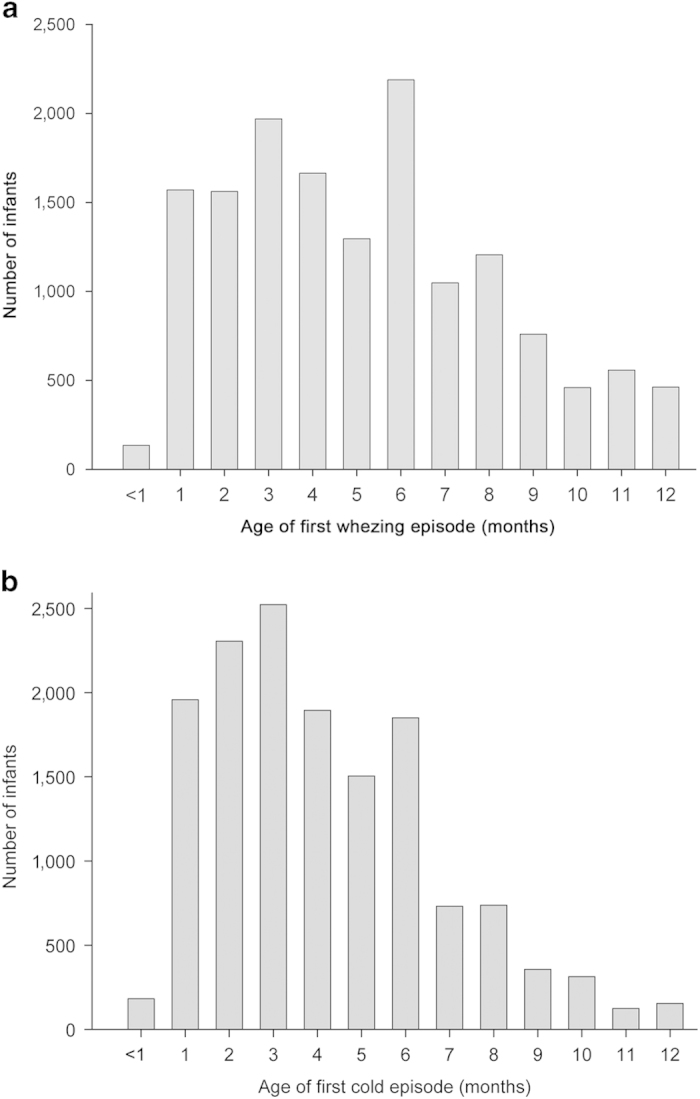
(**a**) Age (months) at which infants had the first episode of wheeze (absolute numbers). (**b**) Age (months) at which infants had the first episode of cold (absolute numbers).

**Figure 2:**
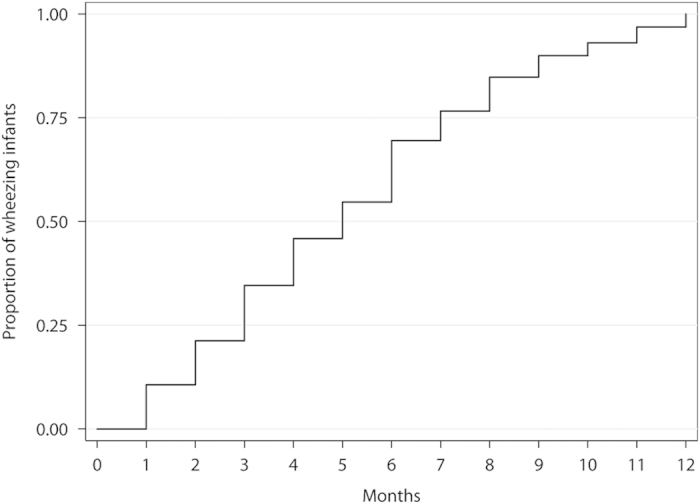
Proportion of wheezing infants by months.

The total number of infants included in the cohort was 35,049 (mean participation rate 83.5%, range 61–91%). Demographic features of the whole population has been published elsewhere.^2^ A total of 15,250 infants had at least one episode of wheeze during the first 12 months of life; however, information on the age (months) when it occurred was not available in 183 of them and these children were excluded from the analysis. Thus, the total number of infants in the analysis was 15,067. Most infants (99.0%) were 18 months or younger when parents were surveyed. Figure 1 shows the distribution by age at which children had their first episode of wheeze, as well as their first cold. In Figure 2 the proportion of wheezing infants by months is represented. In this case the whole population had had an attack of wheezing at the end of the twelfth month. The number of infants included per centre and the proportion of the studied risk/protective factors are shown in Table 1. Missing data of each risk/protective factor by centre are presented in Supplementary Table S1, and the number of children included in this study and those included in the multivariate analysis by centre are detailed in Supplementary Table S2.

Of those factors that have been previously shown to be significantly associated with wheezing in the present cohort,^2^ only male gender (Latin American centres as a group: adjusted hazard ratio (aHR) 1.05, 95% confidence interval (CI) 1.00–1.10, *P*=0.047), parental asthma (all centres as a group: aHR 1.05, 95% CI 1.00–1.11, *P*=0.037), infant eczema (European centres as a group: aHR 1.25, 95% CI 1.12–1.39, *P*<0.001) and having a cold during the first 3 months of life (all centres: aHR 1.97, 95% CI 1.90–2.04, *P*<0.001; and also Latin American: aHR 1.98, 95% CI 1.90–2.06, *P*<0.001; and European: aHR 1.91, 95%CI 1.75–2.09, *P*<0.001 centres as groups) were associated with a shorter period of time to the first wheeze episode. Conversely, exclusive breast feeding for at least 3 months was significantly associated with a longer period of time to the first episode only in LA as a group (aHR 0.91, 95% CI 0.86–0.96, *P*<0.001; Table 2). aHR values of every factor per centre are shown in Supplementary Table S3.

When taking heterogeneity into account, gender and parental asthma showed relatively low inconsistency (<50% in general) both in LA and in EU, although the upper limits of uncertainty intervals were quite high, and comparison between continents showed no differences (*Q*=1.48, *P*=0.224; and *Q*=0.01, *P*=0.936, respectively; Table 2). Having a cold during the first 3 months of life showed high inconsistency (with narrow uncertainty intervals) both in LA and in EU. Although inconsistent on the size of the effect, its direction was invariably towards an association with a shorter time to the first episode (Supplementary Tables S1 and S2). The association of breast feeding with a longer period to the first wheeze episode in LA was quite consistent for centres in that continent (*I* ^2^=35.6%, 95% CI 0–65.6%). The effect of this factor in the European centres was also consistently (*I* ^2^=0%, 95% CI 0–56.7%) non-influential (Supplementary Tables S1 and S2). However, the difference between the summary effects in the two continents reached only marginal significance (*Q*=2.53, *P*=0.112). A similar pattern but in the opposite direction was found for infant eczema: it had a quite consistent association with a shorter period of time to the first wheeze episode in European centres but made no difference at all in Latin American centres. In this case, the difference between continents was highly significant (*Q*=14.3, *P*<0.001).

## Discussion

### Main findings

The results of the present study show that only few factors, of those associated with wheezing in infants during the first years of life, are associated with a shorter time to the first episode of wheeze: having a cold in the first 3 months of life, breast feeding for at least 3 months (but only in Latin American centres) and eczema (only in European centres). Contrarily, very important risk factors for wheeze in the first year of life as found in the same cohort,^2^ such as mother smoking during pregnancy, parental rhinitis, attending a nursery school, number of siblings, number of persons at home and mould stains on household walls, are not factors that apparently shorten the period to the first episode. On the other hand, a consistent protective factor in the Latin American centres,^2^ namely, the mother having undergone university-level education, was not associated with the time to the first wheeze episode.

### Interpretation of findings in relation to previously published work

According to the results of this study the main risk factor to shorten the period to the first wheeze episode is having a cold in the first 3 months of life. Despite the high heterogeneity between the centres, all of them were in the same direction. It seems reasonable—as has also been shown in the present paper—that the earlier the first cold the earlier the first wheeze episode. Certainly, wheeze episodes are mainly triggered by respiratory viruses^20^ that cause symptoms similar to those described in the definition of cold included in the questionnaire used. What our results seem to indicate is that previous colds without wheeze are associated with later wheeze episodes.

It is also of interest that parental asthma, although reaching marginal statistical significance, does not have a substantial influence on the time to the first wheeze episode. In fact, it is only in the Latin American centres that a weak association is found, whereas no association was detected in the European centres. This finding, together with the lack of influence of parental rhinitis, might indicate that the lungs of children born in allergic families behave similarly to those of infants without a family history of asthma and/or rhinitis, at least until the first wheeze episode. In other words, newborn lungs would not necessarily be more prone to suffer from an earlier wheeze episode. Therefore, trying to avoid respiratory infections during the first months of life may be of special importance in those infants with a family history of asthma, as an early episode might have worse consequences than in those infants without that history.^14,16,21,22^

Male gender, like parental asthma, reaches marginal statistical significance only in the Latin American centres; thus, we cannot conclude that it has an important effect on advancing the first wheeze episode. Contrarily, male gender is one of the risk factors reported to be associated with having wheeze episodes during early childhood.^2,8^ This would suggest that, although this respiratory condition is more frequent among boys, gender has no influence on shortening the time to the first wheeze.

According to the present results, exclusive breast feeding for at least 3 months might be a good strategy for delaying the first wheeze episode, but only in Latin American centres. Although our study could not find an association with breast feeding and a longer time to the first wheeze episode in European centres, breast-feeding has been related to reduced wheeze episodes and should be recommended.^2,23^ There is no clear explanation for our results, although it may be related to the type or subtype of viruses that are responsible for triggering the wheeze episodes, which might be diverse in different parts of the world,^24–26^ or to how the same viruses might circulate differently in different areas.^27^ However, breast-feeding has been associated with a lower risk for viral infections in infants.^28^ Cohort studies should be performed in order to elucidate our findings.

Very interestingly, infant eczema was a factor related to a shorter period of time to the first episode of wheezing, but only in European centres. These results, based on the summary HR, are—as breast feeding was—quite consistent among individual centres. In fact, the summary HR difference between the European and the Latin American centres is highly significant. It might be hypothesised that eczema is a marker of atopy and thus predisposes infant lungs to an earlier wheeze episode. This would be a reason for finding that the association only in EU due to asthma is mainly atopic in EU and non-atopic in LA.^29^ However, it is currently not so clear-cut as to what extent atopic eczema is really atopic,^30^ and parental asthma, as another factor for atopy,^31^ does not shorten, in a very significant way, the time to the first episode as discussed previously. On the other hand, atopic eczema has been hypothesised as being related to an imbalance in the skin microbiome,^32^ which might be caused in part by the too frequent use of baths and soaps.^33^ This bathing is probably—as an average—more frequent in EU than in LA. In addition, the present study did not retrieve information as to when the first flare of eczema started and whether it was before or after the first wheeze episode, making this association, found only in EU, difficult to interpret.

### Strengths and limitations of this study

The main strengths of this study are the large number of infants included and the distribution of the centres that agreed to participate.

However, the present study has several limitations. First, it is a cross-sectional study and thus only association and not causality can be inferred from its results. Second, parents were asked about events that had happened during the previous year; therefore, some recall bias could be expected. Conditions such as wheeze, colds or infant eczema were not diagnosed by doctors but reported by parents answering questions describing symptoms. Thus, some misclassification could happen, such as upper airway noises as wheeze. However, questionnaires were validated and are the only feasible way to acquire information from such a numerous sample of infants and have been previously used in large global surveys such as the International Study of Asthma and Allergies in Childhood.^34^ No exclusion criteria were applied to select participants in this study, but conditions that could modify the time to the first wheeze episode, such as prematurity, comorbities (congenital heart conditions, complex syndromes and so on) or admission to a neonatal unit, were not collected. Finally, a high proportion of mothers with university-level education were included in the European centres, mainly because the health centres were in urban areas and this type of population is more prone to collaborate in studies.

### Implications for future research, policy and practice

As the main factor to shorten the time to the first wheeze episode is having a cold in the first 3 months of life, some recommendations could be given to those families with allergy history, which are at higher risk of having wheezing children. These recommendations should be aimed to prevent respiratory infections in their children, mainly by avoiding contact with affected people or avoiding crowded places (for the risk of being infected). Breast feeding should be recommended, although we had found an association with longer time to first wheeze only in LA, as it reduces the risk of an upper respiratory tract infection and severity.^35,36^

## Conclusions

In summary, the main factor for a shorter period of time to the first wheeze episode in infants during their first year of life is having cold symptoms during the first 3 months after birth. Breast feeding for at least 3 months is associated with a later wheeze episode only in Latin American centres, whereas infant eczema is associated with an earlier episode only in European centres. The causes of the differences between continents remain to be elucidated.

## Materials and methods

A detailed description of the EISL study has been published elsewhere previously.^1,2^ In short, all parents of children 11–24 months of age were invited to participate when attending their health-care centres for their child’s immunisations or routine health exams. No exclusion criteria were applied. Parents who accepted answered a retrospective questionnaire of risk factors and symptoms occurring during the child’s first year of life. This study was carried out in 14 centres in LA and in 8 centres in EU (mainly Spain). Samples of at least 1,000 infants were required from participating centres, providing adequate power to detect a statistically significant difference of 5% in the prevalence of recurrent wheeze (three or more episodes) between two centres, with a confidence level of 95%.

### Definitions

Wheezing was defined as a positive answer to the question ‘Has your child had wheezing or whistling in the chest during the first 12 months of his/her life?’, which had been previously validated in the languages of the study.^37–39^ Parental asthma and parental rhinitis were defined as father and/or mother having the disease as reported by them. Infant eczema was reported by parents when answering the question ‘Has your child had an itchy rash, which was coming and going in any area of his/her body, except around the eyes and nose, and the diaper area, during his/her first 12 months of life?’ Common colds were also reported by parents when asked whether their babies had had ‘short episodes of cold with runny nose, sneezing, nasal obstruction, mild cough, with or without mild fever’. In addition, parents were asked about mother smoking during the child’s pregnancy (yes/no); infant attending nursery school during the first year of life (yes/no); exclusive breast feeding (more than 3 versus 3 or fewer months); number of siblings, number of persons living in the same household, existence of mould stains on the walls and presence of pets at the time the questionnaire was taken; maximum study level achieved by the mother (primary/secondary versus university); and ethnicity (Afro-American versus Caucasian).

The outcome variable was defined as the answer to the question ‘At which age has your child had the first episode of wheezing or whistling in the chest?’ (in months).

### Data analysis

Known statistically significant risk factors for wheezing in the first year of life in the present cohort^2^ and defined above were tested for their association with a shorter or longer period of time from birth to the first wheeze episode. With respect to smoking, a high colineality between smoking during pregnancy by the mother and afterwards was found; thus, only smoking in pregnancy was included in the analyses. This was performed within each centre by means of a survival analysis, using the Cox proportional hazards model test, in order to obtain HR and their respective 95% CI (Stata v7, College Station, TX, USA). A crude HR and an aHR for the remaining factors were calculated for each factor. Only those infants having at least one episode of wheezing were entered in this analysis, as our aim was to know the factors associated with a shorter time to the first wheeze episode, and thus no interest existed in including those who did not wheeze. Moreover, a previous analysis including the whole sample (those who wheezed and those who did not) was performed in order to know the factors associated with wheezing,^2^ which were the factors tested in the current analysis. Ethnicity was only included in those centres with at least 5% of the individuals being of Afro-American origin (centres from Brazil, Colombia, El Salvador and Honduras).

Summary HR and aHR with 95% CIs were calculated using the meta-analysis approach with random effects in order to allow for the weight of the centre^40^ by means of CMA v2.2 software (Borenstein M, Hedges L, Higgins J, Rothstein H; Comprehensive Meta-analysis Version 2, Biostat, Englewood, NJ, USA, 2005). Summary aHRs were calculated for all centres and also for centres of LA and EU separately, and differences between continents were compared by means of the Cochran *Q* test. The *I* ^2^ statistic was used to assess the percentage of heterogeneity for every summary aHR. Twenty-five, fifty and seventy-five percent were considered as cutoff points for low, medium and high heterogeneity, respectively.^41^ A Kaplan–Meier curve was performed to represent the proportion of wheezing infants by age.

### Ethical approval

The study was approved by the local Scientific Ethic Committee at each centre, and parents or guardians answered the questionnaire after providing their fully informed written consent. The data set was anonymised.

## Figures and Tables

**Table 1:** Population of wheezing children (at least one episode) in the first year of life, together with the prevalence of risk or protective factors (%) by centre^a^

	*Sample size*	*Male gender*	*Parental asthma*	*Parental rhinitis*	*Infant eczema*	*Smoking in pregnancy*	*Cold(s) in first 3 months*	*Attending nursery school*	*Breastfed 3+ months*	*Siblings med* *(IQR)*	*Persons at home med* *(IQR)*	*Mould stains*	*University studies in mother*	*Afro-American ethnicity*	*Pets at home*
*Chile*															
Santiago	1,761	52.4	18.8	23.0	49.5	10.7	53.7	9.5	77.6	1 (0–2)	5 (4–7)	26.5	57.2	0	55.3
Valdivia	1,674	60.3	24.6	30.7	53.3	9.6	49.5	20.2	72.4	1 (0–2)	4 (3–5)	36.8	59.6	0	45.0

*Brazil*															
Fortaleza	546	57.3	46.1	48.7	61.5	12.8	42.2	3.7	57.1	1 (0–2)	4 (3–6)	19.7	20.7	52.1	36.8
Recife	457	52.5	48.1	39.8	64.3	15.3	49.9	34.1	59.5	1 (0–2)	4 (3–6)	33.3	25.6	60.0	29.5
Belo Horizonte	1,265	54.5	49.0	58.0	63.9	15.7	45.0	15.1	59.5	1 (0–2)	4 (3–5)	40.2	32.1	47.5	49.0
Belem	1,395	54.0	23.6	35.9	66.0	6.2	48.1	1.8	78.3	1 (0–2)	5 (4–7)	29.5	26.6	64.7	46.2
Porto Alegre	643	54.7	44.2	68.3	63.7	22.7	36.9	25.0	92.5	1 (0–2)	4 (3–5)	44.6	2.8	28.3	52.1
São Paulo	465	55.7	18.7	54.4	51.4	21.3	43.5	26.4	59.1	1 (0–2)	4 (3–5)	39.3	38.7	33.1	34.4
Curitiba	1,354	54.6	26.0	61.8	59.3	15.4	46.5	31.6	66.6	1 (0–2)	4 (3–5)	35.5	36.5	14.5	51.3
															
*Colombia*															
Barranquilla	776	54.4	35.7	54.9	59.8	5.2	50.3	7.5	67.2	1 (0–3)	6 (5–8)	51.2	31.6	7.6	44.0

*Mexico*															
Mérida	176	60.8	30.1	50.0	21.6	1.7	2.3	63.6	44.9	1 (1–2)	5 (4–6)	26.7	43.7	0	48.9
															
*Venezuela*															
Caracas	1,223	55.9	52.6	52.5	47.7	8.6	53.2	20.4	54.4	1 (0–2)	5 (4–7)	32.1	45.6	0	38.3

*El Salvador*															
La Libertad	428	54.2	17.2	33.3	15.7	1.2	50.5	8.2	67.7	1 (0–2)	4 (4–6)	26.6	43.8	8.3	47.9
															
*Honduras*															
S Pedro Sula	215	61.8	34.0	35.4	27.1	3.7	58.1	1.4	31.6	1 (0–2)	4 (3–6)	22.2	13.1	9.8	31.3

Latin America total	12,202	55.3	32.6	44.8	55.3	11.4	48.6	16.0	68.2	1 (0–2)	5 (3–6)	34.1	38.6	22.2	45.8

*Spain*															
Valencia	249	56.9	14.5	31.7	16.7	18.4	40.2	24.0	43.0	1 (0–1)	4 (3–4)	3.6	74.6	0	25.3
Cartagena	453	61.1	19.2	25.5	18.7	26.5	42.3	15.4	36.5	1 (0–1)	4 (3–4)	17.7	72.1	0.4	29.5
Bilbao	384	55.7	16.9	25.1	15.8	20.8	28.9	39.7	49.2	1 (0–1)	4 (3–4)	10.5	91.1	0	11.0
La Coruña	316	60.4	20.0	31.3	19.2	25.9	38.4	32.4	40.4	1 (0–1)	3 (3–4)	15.2	86.3	0.3	25.6
Salamanca	365	54.2	9.6	22.8	21.4	14.2	41.9	35.3	46.8	1 (0–1)	4 (3–4)	9.6	87.1	2.7	20.3
Cantabria	303	57.4	24.2	32.2	16.9	20.7	45.8	28.1	42.9	1 (0–1)	4 (3–4)	20.3	88.0	4.0	24.7
Pamplona	300	61.7	16.5	27.6	18.0	20.2	28.9	43.7	56.8	1 (0–1)	3 (3–4)	5.4	88.7	2.7	18.8
															
*The Netherlands*															
Zwolle	308	58.4	20.1	56.2	30.7	7.6	58.2	48.4	44.3	1 (0–1)	3 (3–4)	7.5	98.7	0.6	59.1

Europe total	2,865	58.4	18.3	32.1	19.8	18.5	38.0	34.7	44.7	1 (0–1)	4 (3–4)	12.6	82.9	1.3	27.8
Total	15,067	55.9	29.9	42.4	48.6	12.7	46.6	19.6	63.7	1 (0–2)	4 (3–6)	30.1	47.0	18.4	42.4

Abbreviations: IQR, interquartile range; med, median.

a Missing data in a specific variable not included in the calculation of percentage in each factor.

**Table 2:** Summary aHRs (95% CIs) and *P* values for the different risk or protective factors as calculated on all centres or on centres grouped by continent according to meta-analyses of the results obtained in each centre

	*All centres*			*Latin American centres*			*European centres*			*LA* *versus EU*	
	*Summary aHR*	P-*value*	I^*2*^ *(95% CI)*	*Summary aHR*	P-*value*	I^*2*^ *(95% CI)*	*Summary aHR*	P-*value*	I^*2*^ *(95% CI)*	Q	P-*value*
Male gender	1.03 (0.97–1.09)	0.339	23.5 (0–54.6)	1.05 (1.00–1.10)	0.047	25.6 (0–57.8)	0.98 (0.89–1.08)	0.722	21.0 (0–66.3)	1.48	0.224
Parental asthma	1.05 (1.00–1.11)	0.037	19.6 (0–52.1)	1.06 (1.00–1.12)	0.051	0 (0–49.8)	1.05 (0.92–1.19)	0.455	50.4 (0–77.8)	0.01	0.936
Parental rhinitis	1.03 (0.99–1.08)	0.118	4.6 (0–35.8)	1.03 (0.99–1.08)	0.178	0 (0–26.7)	1.04 (0.94–1.16	0.413	49.1 (0.77.3)	0.04	0.839
Infant eczema	1.11 (0.89–1.38)	0.365	20.4 (0–52.6)	0.99 (0.96–1.04)	0.818	0 (0–0)	1.25 (1.12–1.39)	<0.001	6.7 (0–43.9)	14.3	<0.001
Mother smoked in pregnancy	1.03 (0.95–1.11)	0.488	8.7 (0–42.9)	1.00 (0.93–1.07)	0.995	0 (0–52.6)	1.08 (0.97–1.22)	0.170	19.7 (0–62.2)	1.40	0.236
Cold during first 3 months	1.97 (1.79–2.17)	<0.001	82.7 (74.9–88.1)	1.98 (1.76–2.22)	<0.001	85.6 (77.3–90.8)	1.96 (1.66–2.32)	<0.001	77.7 (55.9–88.7.7)	0.01	0.945
Attending nursery school	0.97 (0.90–1.05)	0.493	6.6 (0–40.0)	1.00 (0.95–1.06)	0.884	0 (0–49.8)	0.93 (0.84–1.02)	0.111	16.5 (0–59.5)	2.09	0.148
Breast feeding >3 months	0.94 (0.86–1.02)	0.141	26.9 (0–56.6)	0.91 (0.86–0.96)	<0.001	35.6 (0–65.6)	0.99 (0.90–1.09)	0.837	0 (0–56.7)	2.53	0.112
Per additional sibling	1.01 (0.99–1.03)	0.385	40.3 (0.48–64.1)	1.01 (0.99–1.03)	0.436	27.9 (0–61.9)	1.02 (0.94–1.10)	0.676	59.0 (10.6–81.2)	0.03	0.865
Per additional person at home	1.00 (0.99–1.02)	0.782	44.8 (8.8–66.6)	1.00 (0.99–1.02)	0.660	51.7 (11.1–73.8)	0.99 (0.95–1.03)	0.681	33.2 (0–70.4)	0.29	0.593
Mould stains	1.04 (0.98–1.10)	0.182	33.6 (0–60.4)	1.05 (0.99–1.11)	0.145	36.1 (0–66.2)	1.00 (0.86–1.16)	0.959	34.6 (0–71.0)	0.35	0.554
University studies in mother	0.97 (0.92–1.02)	0.246	17.8 (0–50.8)	0.96 (0.92–1.02)	0.258	22.3 (0–58.5)	0.98 (0.85–1.12)	0.767	20.5 (0–62.9)	0.02	0.887
Afro-American ethnicity	0.97 (0.92–1.02)	0.253	0 (0–48.7)	0.97 (0.92–1.02)	0.257	0 (0–54.1)	NA	—	—	NA	—
Pets at home	0.99 (0.93–1.06)	0.806	56.8 (30.4–73.2)	1.00 (0.93–1.08)	0.914	65.2 (38.7–80.3)	0.96 (0.84–1.09)	0.489	33.6 (0–70.4)	0.43	0.512

Comparison between Latin American and European countries is expressed as the *Q* statistic and its corresponding *P* value.

Heterogeneity is indicated as *I*^2^ (95% CIs).

Abbreviations: aHR, adjusted hazard ratio; EU, Europe; LA, Latin America; NA, non-applicable as the Afro-American population was <5% in all centres.

## EISL* Study Group

Álvaro Madeiro Leite, Olivia Costa Bessa (Brazil, Fortaleza); Elaine Xavier Prestes (Brazil, Belem); Emanuel Sarinho, Décio Medeiros (Brazil, Recife); Paulo Camargos, Maria Jussara Fernández-Fontes, Wilson Rocha (Brazil, Bello Horizonte); Dirceu Solé, Caroline Della Bianca (Brazil, São Paulo); Nelson Rosario, Herberto Chong (Brazil, Curitiba); Gilberto B Fischer (Brazil, Porto Alegre); Eduardo Cepeda (Colombia, Barranquilla); Oscar Aldrey, Arnaldo Capriles (Venezuela, Caracas); Javier Mallol, Viviana Aguirre, Alejandro Gallardo (Chile, Santiago); Mario Calvo (Chile, Valdivia); Manuel Baeza-Bacab, (México, Mérida); Margarita Figueroa (La Libertad, El Salvador); Agustín Bueso, Luis Cousin (San Pedro Sula, Honduras); Luis Garcia-Marcos, Antonela Martinez-Torres, Virginia Pérez-Fernández, Manuel Sánchez-Solís, Maria Sánchez-Bahillo (España, Murcia), Carlos González-Díaz, Andrés González Hermosa (España, Bilbao), Angel López-Silvarrey Varela (España, La Coruña), María Morales Suárez-Varela (España, Valencia); Paul LP Brand, Chantal AN Visser (Zwolle, The Netherlands).

*EISL for ‘Estudio Internacional de Sibilancias en Lactantes’ (International Study of Wheezing in Infants).

## Notes

Supplementary Table 1S

Supplementary Table 2S

Supplementary Table 3S
