# Supplementary material for: Factors associated with the time to the first wheezing episode in infants: a cross-sectional study from the International Study of Wheezing in Infants (EISL)
Source: NPJ Prim Care Respir Med. 2016 Jan 21;26:15077–. doi: 10.1038/npjpcrm.2015.77 (PMC4721498; doi:10.1038/npjpcrm.2015.77)
Supplement: Supplementary Table 1S [file npjpcrm201577-s1.doc]

Table 1S. Population of wheezing children (at least one episode in the first year of life), together with the percentage of missing data of each risk or protective factor by centre.

|  | Sample  size | Male  gender | Parental  asthma | Parental  rhinitis | Infant  eczema | Smoking  in  pregnancy | Cold(s in  first 3  months | Attending  nursery  school | Breastfed  3+ months | Siblings | Persons  at home | Mould  stains | University  studies  in mother | Afro-  american  ethnicity | Pets at  home |
| --- | --- | --- | --- | --- | --- | --- | --- | --- | --- | --- | --- | --- | --- | --- | --- |
| *Chile* |  |  |  |  |  |  |  |  |  |  |  |  |  |  |  |
| Santiago | 1761 | 0.2 | 11.1 | 8.5 | 2.3 | 1.6 | 2.9 | 2.2 | 5.8 | 6.9 | 1.9 | 2.7 | 5.6 | 100 | 6.2 |
| Valdivia | 1674 | 0.1 | 0 | 0 | 0 | 0 | 0 | 0 | 0 | 0 | 0 | 0 | 0 | 0 | 0 |
| *Brazil* |  |  |  |  |  |  |  |  |  |  |  |  |  |  |  |
| Fortaleza | 546 | 0 | 0.2 | 0 | 0.2 | 0 | 4.6 | 0 | 0.2 | 0.5 | 0.2 | 1.6 | 0 | 0.4 | 0 |
| Recife | 457 | 0 | 0 | 0 | 0 | 0 | 4.8 | 0 | 0.7 | 0 | 0 | 0 | 0 | 0 | 0 |
| Belo Horizonte | 1265 | 0 | 0.4 | 2.2 | 0.2 | 0.1 | 2.1 | 0.2 | 0.5 | 1.1 | 0.4 | 0.1 | 0.5 | 2.3 | 0.2 |
| Belem | 1395 | 0 | 0 | 0 | 0 | 0 | 0 | 0 | 0 | 0 | 0 | 0 | 0 | 0 | 0 |
| Porto Alegre | 643 | 0 | 0 | 0.2 | 0.5 | 0.3 | 6.8 | 0.5 | 0.8 | 0.9 | 0.6 | 0.9 | 1.2 | 0 | 0.3 |
| Sao Paulo | 465 | 0 | 0 | 0 | 0.4 | 0 | 4.1 | 0 | 1.7 | 0.2 | 0.2 | 0.9 | 0 | 0.2 | 0 |
| Curitiba | 1354 | 0 | 1.0 | 0.7 | 1.1 | 0.6 | 7.4 | 0.7 | 3.2 | 14.2 | 0.4 | 0.7 | 1.5 | 0.9 | 0.7 |
| *Colombia* |  |  |  |  |  |  |  |  |  |  |  |  |  |  |  |
| Barranquilla | 776 | 0.1 | 0.1 | 0.1 | 0.4 | 0.4 | 2.3 | 1.7 | 1.7 | 0 | 0 | 0.5 | 1.4 | 1.5 | 9.8 |
| *Mexico* |  |  |  |  |  |  |  |  |  |  |  |  |  |  |  |
| Mérida | 176 | 0 | 0 | 0 | 0 | 0 | 0 | 0 | 0 | 0 | 0 | 0 | 0 | 100 | 0 |
| *Venezuela* |  |  |  |  |  |  |  |  |  |  |  |  |  |  |  |
| Caracas | 1223 | 0.1 | 0 | 0 | 0 | 0.2 | 1.6 | 0 | 6.8 | 0.8 | 0.7 | 0.4 | 0.2 | 100 | 0 |
| *El Salvador* |  |  |  |  |  |  |  |  |  |  |  |  |  |  |  |
| La Libertad | 428 | 0 | 0.9 | 0.2 | 0.2 | 0 | 0 | 0.7 | 0 | 0 | 0.2 | 0.7 | 0.2 | 1.4 | 1.4 |
| *Honduras* |  |  |  |  |  |  |  |  |  |  |  |  |  |  |  |
| S Pedro Sula | 215 | 0 | 1.4 | 1.4 | 0.5 | 0 | 0 | 0.5 | 0 | 0 | 0 | 1.4 | 0.9 | 5.1 | 0.5 |
|  |  |  |  |  |  |  |  |  |  |  |  |  |  |  |  |
| **Latin America Total** | **12202** | **0.1** | **1.8** | **1.6** | **0.6** | **0.4** | **2.7** | **0.6** | **2.2** | **2.8** | **0.5** | **0.7** | **1.2** | **25.0** | **1.7** |
|  |  |  |  |  |  |  |  |  |  |  |  |  |  |  |  |
| *Spain* |  |  |  |  |  |  |  |  |  |  |  |  |  |  |  |
| Valencia | 249 | 2.4 | 1.2 | 1.2 | 0.4 | 0 | 3.2 | 0.8 | 0.4 | 0.4 | 0 | 1.6 | 0 | 5.2 | 1.2 |
| Cartagena | 453 | 0 | 3.3 | 4.0 | 0.9 | 0.9 | 4.0 | 1.1 | 2.0 | 1.5 | 1.1 | 1.8 | 3.3 | 5.1 | 6.4 |
| Bilbao | 384 | 0 | 1.3 | 1.6 | 1.3 | 0.5 | 2.9 | 0.3 | 0 | 0 | 0 | 0.8 | 0.5 | 3.4 | 0.3 |
| La Coruña | 316 | 0.9 | 2.8 | 2.8 | 3.4 | 0.6 | 8.5 | 0.3 | 2.2 | 1.2 | 0.9 | 0.9 | 4.1 | 1.9 | 3.4 |
| Salamanca | 365 | 0 | 0.5 | 0.3 | 0.3 | 0 | 0 | 0 | 0 | 0 | 0 | 0 | 0 | 0 | 0.3 |
| Cantabria | 303 | 0.3 | 2.0 | 3.0 | 1.0 | 0.7 | 1.0 | 0.7 | 0.3 | 0 | 0 | 1.0 | 1.0 | 0.7 | 1.3 |
| Pamplona | 300 | 1.3 | 6.2 | 5.9 | 3.3 | 2.0 | 0 | 1.3 | 0.3 | 0 | 0 | 2.0 | 0.7 | 1.0 | 2.0 |
| *The Netherlands* |  |  |  |  |  |  |  |  |  |  |  |  |  |  |  |
| Zwolle | 308 | 0 | 0 | 0 | 0.6 | 0.3 | 3.6 | 0.6 | 0.3 | 0.6 | 0.6 | 0.6 | 1.3 | 5.5 | 0 |
|  |  |  |  |  |  |  |  |  |  |  |  |  |  |  |  |
| **Europe Total** | **2865** | **0.5** | **2.1** | **2.2** | **1.3** | **0.6** | **2.7** | **0.6** | **0.7** | **0.5** | **0.3** | **1.0** | **1.4** | **8.8** | **1.9** |
|  |  |  |  |  |  |  |  |  |  |  |  |  |  |  |  |
| **Total** | **15067** | **0.1** | **1.9** | **1.7** | **0.7** | **0.4** | **2.7** | **0.6** | **1.9** | **2.4** | **0.5** | **0.8** | **1.2** | **22.0** | **1.7** |
