# Supplementary material for: Factors associated with the time to the first wheezing episode in infants: a cross-sectional study from the International Study of Wheezing in Infants (EISL)
Source: NPJ Prim Care Respir Med. 2016 Jan 21;26:15077–. doi: 10.1038/npjpcrm.2015.77 (PMC4721498; doi:10.1038/npjpcrm.2015.77)
Supplement: Supplementary Table 2S [file npjpcrm201577-s2.doc]

Table 2S. Total population of wheezing children (at least one episode) in the first year of life and number of infants included in the multivariate analysis by centre.

|  | Total  sample | Included  infants |
| --- | --- | --- |
| *Chile* |  |  |
| Santiago | 1761 | 1179 |
| Valdivia | 1674 | 1672 |
| *Brazil* |  |  |
| Fortaleza | 546 | 505 |
| Recife | 457 | 401 |
| Belo Horizonte | 1265 | 1151 |
| Belem | 1395 | 1395 |
| Porto Alegre | 643 | 566 |
| Sao Paulo | 465 | 423 |
| Curitiba | 1354 | 991 |
| *Colombia* |  |  |
| Barranquilla | 776 | 635 |
| *Mexico* |  |  |
| Mérida | 176 | 176 |
| *Venezuela* |  |  |
| Caracas | 1223 | 1079 |
| *El Salvador* |  |  |
| La Libertad | 428 | 398 |
| *Honduras* |  |  |
| S Pedro Sula | 215 | 193 |
|  |  |  |
| **Latin America Total** | **12202** | **10764** |
|  |  |  |
| *Spain* |  |  |
| Valencia | 249 | 222 |
| Cartagena | 453 | 356 |
| Bilbao | 384 | 355 |
| La Coruña | 316 | 236 |
| Salamanca | 365 | 361 |
| Cantabria | 303 | 269 |
| Pamplona | 300 | 243 |
| *The Netherlands* |  |  |
| Zwolle | 308 | 281 |
|  |  |  |
| **Europe Total** | **2865** | **2323** |
|  |  |  |
| **Total** | **15067** | **13087** |
