# Supplementary material for: Factors associated with the time to the first wheezing episode in infants: a cross-sectional study from the International Study of Wheezing in Infants (EISL)
Source: NPJ Prim Care Respir Med. 2016 Jan 21;26:15077–. doi: 10.1038/npjpcrm.2015.77 (PMC4721498; doi:10.1038/npjpcrm.2015.77)
Supplement: Supplementary Table 3S [file npjpcrm201577-s3.doc]

Table 3S. Adjusted^1^ hazard ratios of each risk/protective factor per centre.

|  | Male  Gender | Family history | | Infant  eczema | Mother  smoked  during  pregnancy | Colds  during  first 3  months | Attending  nursery  school | Breast  feeding  >3m | Per  additional  Sibling | Per  additional  persons  at home | Mould  stains | University  studies  in mother | Afro-  American  ethnicity | Pets at  home |
| --- | --- | --- | --- | --- | --- | --- | --- | --- | --- | --- | --- | --- | --- | --- |
|  |  | Asthma | Rhinitis |  |  |  |  |  |  |  |  |  |  |  |
| Chile |  |  |  |  |  |  |  |  |  |  |  |  |  |  |
| Santiago | 1.13  (1.00-1.27) | 0.98  (0.84-1.14) | 0.98  (0.85-1.12) | 0.98  (0.87-1.10) | 0.80  (0.66-0.97) | 2.52  (2.23-2.85) | 1.15  (0.95-1.39) | 0.86  (0.75-0.99) | 1.08  (1.03-1.13) | 1.02  (1.00-1.05) | 0.87  (0.76-1.00) | 0.88  (0.78-1.00) | NA | 0.91  (0.81-1.03) |
| Valdivia | 0.93  (0.84-1.02) | 1.06  (0.94-1.20) | 1.08  (0.96-1.21) | 0.99  (0.90-1.09) | 0.99  (0.83-1.18) | 2.35  (2.12-2.61) | 1.03  (0.91-1.18) | 1.07  (0.95-1.19) | 1.02  (0.98-1.08) | 0.94  (0.91-0.97) | 1.20  (1.07-1.35) | 1.06  (0.94-1.18) | NA | 1.26  (1.14-1.40) |
| Brazil |  |  |  |  |  |  |  |  |  |  |  |  |  |  |
| Fortaleza | 1.03  (0.86-1.24) | 1.16  (0.97-1.39) | 0.97  (0.81-1.16) | 0.96  (0.80-1.16) | 1.04  (0.79-1.37) | 1.84  (1.53-2.21) | 1.56  (0.98-2.48) | 1.08  (0.90-1.29) | 0.98  (0.92-1.05) | 1.00  (0.97-1.04) | 1.12  (0.89-1.40) | 1.12  (0.89-1.40) | 1.05  (0.88-1.26) | 1.09  (0.91-1.32) |
| Recife | 1.10  (0.90-1.35) | 0.98  (0.80-1.21) | 1.12  (0.90-1.38) | 1.00  (0.81-1.24) | 0.90  (0.67-1.21) | 2.00  (1.62-2.45) | 0.93  (0.73-1.18) | 0.77  (0.63-0.95) | 1.00  (0.93-1.08) | 0.99  (0.95-1.04) | 1.09  (0.88-1.36) | 0.98  (0.77-1.25) | 0.95  (0.76-1.17) | 0.83  (0.66-1.03) |
| Belo Horizonte | 1.00  (0.89-1.13) | 1.06  (0.94-1.20) | 1.07  (0.95-1.21) | 1.02  (0.90-1.16) | 1.08  (0.91-1.28) | 1.84  (1.63-2.07) | 1.09  (0.92-1.28) | 0.88  (0.78-0.99) | 0.99  (0.94-1.03) | 1.02  (0.98-1.05) | 1.06  (0.93-1.20) | 0.99  (0.87-1.13) | 0.95  (0.84-1.07) | 0.97  (0.86-1.09) |
| Belem | 1.01  (0.91-1.12) | 1.02  (0.90-1.16) | 1.02  (0.91-1.14) | 0.95  (0.85-1.06) | 0.99  (0.79-1.24) | 2.08  (1.87-2.32) | 0.90  (0.60-1.34) | 0.87  (0.77-1.00) | 0.98  (0.93-1.02) | 1.01  (0.99-1.04) | 1.03  (0.91-1.15) | 0.95  (0.84-1.07) | 0.99  (0.89-1.11) | 0.92  (0.83-1.03) |
| Porto Alegre | 1.08  (0.91-1.27) | 1.04  (0.87-1.24) | 1.04  (0.86-1.25) | 1.10  (0.92-1.32) | 1.12  (0.91-1.38) | 0.77  (0.65-0.92) | 0.96  (0.79-1.16) | 1.00  (0.72-1.39) | 1.05  (0.99-1.11) | 1.04  (0.99-1.09) | 0.90  (0.76-1.07) | 1.03  (0.61-1.72) | 1.00  (0.82-1.21) | 0.96  (0.81-1.14) |
| Sao Paulo | 1.06  (0.87-1.30) | 1.19  (0.93-1.53) | 0.98  (0.80-1.20) | 0.99  (0.81-1.21) | 0.91  (0.71-1.16) | 2.18  (1.78-2.68) | 0.93  (0.74-1.16) | 1.01  (0.83-1.23) | 1.04  (0.97-1.12) | 0.95  (0.90-1.01) | 1.06  (0.86-1.29) | 0.83  (0.68-1.03) | 0.89  (0.72-1.10) | 1.22  (0.99-1.51) |
| Curitiba | 1.24  (1.09-1.41) | 1.17  (1.01-1.36) | 1.02  (0.89-1.17) | 0.96  (0.84-1.09) | 1.06  (0.89-1.27) | 1.77  (1.55-2.01) | 0.96  (0.84-1.11) | 0.83  (0.72-0.95) | 1.02  (0.96-1.08) | 1.02  (0.98-1.07) | 1.09  (0.95-1.24) | 0.94  (0.81-1.08) | 1.09  (0.91-1.30) | 1.00  (0.88-1.14) |
| Colombia |  |  |  |  |  |  |  |  |  |  |  |  |  |  |
| Barranquilla | 1.12  (0.95-1.31) | 1.00  (0.83-1.19) | 0.96  (0.80-1.14) | 1.02  (0.86-1.20) | 1.45  (0.99-2.12) | 1.85  (1.57-2.18) | 1.13  (0.80-1.60) | 0.85  (0.71-1.01) | 1.01  (0.97-1.06) | 1.00  (0.98-1.03) | 1.11  (0.94-1.31) | 0.87  (0.73-1.03) | 1.06  (0.78-1.43) | 0.85  (0.72-1.00) |
| Mexico |  |  |  |  |  |  |  |  |  |  |  |  |  |  |
| Mérida | 1.04  (0.76-1.43) | 1.21  (0.85-1.70) | 0.86  (0.63-1.18) | 1.34  (0.57-3.17) | 0.88  (0.27-2.90) | 94.17  (21.90-404.90) | 0.96  (0.69-1.33) | 0.89  (0.63-1.25) | 0.89  (0.67-1.20) | 1.04  (0.85-1.27) | 0.74  (0.33-1.64) | 1.13  (0.81-1.56) | NA | 0.88  (0.63-1.22) |
| Venezuela |  |  |  |  |  |  |  |  |  |  |  |  |  |  |
| Caracas | 1.00  (0.88-1.13) | 1.12  (0.99-1.27) | 1.11  (0.98-1.26) | 0.98  (0.87-1.11) | 0.95  (0.77-1.18) | 1.69  (1.50-1.91) | 0.89  (0.76-1.03) | 0.92  (0.81-1.04) | 0.97  (0.93-1.02) | 1.00  (0.98-1.02) | 1.12  (0.98-1.27) | 0.99  (0.87-1.12) | NA | 1.06  (0.94-1.20) |
| El Salvador |  |  |  |  |  |  |  |  |  |  |  |  |  |  |
| La Libertad | 1.02  (0.83-1.26) | 0.79  (0.59-1.06) | 1.13  (0.90-1.43) | 1.15  (0.87-1.52) | 0.82  (0.32-2.10) | 1.75  (1.41-2.16) | 1.16  (0.80-1.68) | 0.80  (0.64-1.01) | 0.99  (0.92-1.06) | 1.02  (0.98-1.07) | 1.05  (0.82-1.33) | 0.91  (0.73-1.14) | 1.21  (0.82-1.77) | 1.05  (0.86-1.28) |
| Honduras |  |  |  |  |  |  |  |  |  |  |  |  |  |  |
| San Pedro Sula | 1.02  (0.75-1.39) | 0.92  (0.67-1.25) | 0.85  (0.62-1.16) | 1.11  (0.78-1.57) | 1.14  (0.49-2.68) | 2.33  (1.68-3.24) | 1.13  (0.26-4.82) | 0.87  (0.63-1.18) | 0.98  (0.86-1.12) | 1.00  (0.91-1.10) | 0.84  (0.58-1.20) | 1.66  (1.06-2.60) | 0.74  (0.44-1.22) | 1.11  (0.79-1.54) |
| Spain |  |  |  |  |  |  |  |  |  |  |  |  |  |  |
| Valencia | 0.94  (0.71-1.24) | 1.79  (1.16-2.77) | 0.98  (0.70-1.37) | 0.94  (0.64-1.40) | 1.13  (0.77-1.65) | 3.26  (2.42-4.39) | 0.75  (0.54-1.05) | 1.00  (0.75-1.32) | 0.96  (0.83-1.12) | 0.97  (0.80-1.18) | 0.84  (0.37-1.90) | 0.70  (0.49-0.99) | NA | 0.66  (0.48-0.92) |
| Cartagena | 0.76  (0.61-0.95) | 1.08  (0.80-1.47) | 1.07  (0.82-1.40) | 1.31  (1.00-1.74) | 1.15  (0.90-1.46) | 1.99  (1.59-2.50) | 0.96  (0.70-1.31) | 0.97  (0.77-1.22) | 0.99  (0.84-1.16) | 1.11  (0.98-1.25) | 1.00  (0.75-1.34) | 0.94  (0.74-1.20) | NA | 1.14  (0.90-1.44) |
| Bilbao | 0.98  (0.78-1.22) | 1.06  (0.77-1.45) | 1.10  (0.85-1.42) | 1.59  (1.17-2.15) | 1.39  (1.05-1.82) | 1.28  (1.01-1.63) | 1.02  (0.81-1.28) | 1.19  (0.95-1.49) | 1.07  (0.84-1.37) | 0.87  (0.69-1.08) | 1.07  (0.75-1.52) | 0.91  (0.61-1.37) | NA | 0.82  (0.57-1.18) |
| La Coruña | 1.14  (0.86-1.52) | 0.95  (0.66-1.36) | 0.79  (0.58-1.07) | 1.27  (0.90-1.79) | 0.96  (0.69-1.33) | 2.36  (1.78-3.13) | 0.93  (0.71-1.24) | 0.99  (0.75-1.31) | 1.03  (0.82-1.28) | 0.97  (0.92-1.03) | 1.16  (0.80-1.69) | 0.63  (0.43-0.94) | NA | 0.97  (0.72-1.32) |
| Salamanca | 1.09  (0.88-1.37) | 0.77  (0.52-1.16) | 1.54  (1.17-2.02) | 1.18  (0.90-1.55) | 0.80  (0.57-1.10) | 1.87  (1.49-2.34) | 0.73  (0.57-0.94) | 0.98  (0.78-1.24) | 0.71  (0.58-0.87) | 0.98  (0.90-1.07) | 1.29  (0.86-1.96) | 0.99  (0.70-1.41) | NA | 0.83  (0.62-1.10) |
| Cantabria | 1.01  (0.78-1.30) | 0.93  (0.68-1.26) | 0.88  (0.66-1.16) | 1.49  (1.06-2.08) | 1.12  (0.82-1.53) | 1.67  (1.30-2.14) | 1.12  (0.85-1.48) | 0.87  (0.68-1.13) | 1.11  (0.90-1.37) | 1.13  (0.98-1.31) | 0.71  (0.52-0.97) | 1.49  (0.98-2.25) | NA | 1.14  (0.86-1.51) |
| Pamplona | 0.92  (0.70-1.20) | 0.77  (0.51-1.14) | 1.18  (0.84-1.65) | 0.99  (0.70-1.40) | 1.14  (0.82-1.60) | 2.56  (1.88-3.48) | 0.86  (0.66-1.13) | 1.09  (0.83-1.43) | 1.28  (0.98-1.68) | 0.87  (0.73-1.05) | 0.60  (0.31-1.16) | 1.02  (0.64-1.63) | NA | 1.02  (0.73-1.42) |
| The Netherlands |  |  |  |  |  |  |  |  |  |  |  |  |  |  |
| Zwolle | 1.16  (0.90-1.49) | 1.37  (1.00-1.87) | 0.95  (0.74-1.22) | 1.20  (0.91-1.57) | 0.83  (0.51-1.34) | 1.60  (1.24-2.07) | 1.03  (0.80-1.32) | 0.84  (0.65-1.09) | 1.11  (0.94-1.30) | 0.97  (0.83-1.13) | 1.33  (0.82-2.16) | 0.73  (0.23-2.35) | NA | 1.04  (0.81-1.34) |

^1^adjusted for all factors in the table.

NA: non-applicable as Afro-American population was less than 5%.
